# Clinical and imaging features of carcinosarcoma of the uterus and cervix

**DOI:** 10.1186/s13244-021-01084-5

**Published:** 2021-10-21

**Authors:** Liming Li, Wenpeng Huang, Kangkang Xue, Leiyu Feng, Yijing Han, Rui Wang, Jianbo Gao

**Affiliations:** 1 Department of Radiology, The First Affiliated Hospital of Zhengzhou University, No. 1, East Jianshe Road, Zhengzhou, 450052 Henan China; 2 Department of Magnetic Resonance Imaging, The First Affiliated Hospital of Zhengzhou University, No. 1, East Jianshe Road, Zhengzhou, 450052 Henan China; 3 Department of Internal Medicine, The First Affiliated Hospital of Zhengzhou University, No. 1, East Jianshe Road, Zhengzhou, 450052 Henan China

**Keywords:** Uterine carcinosarcoma, Cervix, Tomography (X-ray computed), Magnetic resonance imaging, Diagnosis

## Abstract

**Aim:**

The purpose of our study was to analyze the clinical and imaging features of uterine carcinosarcoma (UCS) and cervical carcinosarcoma (CCS), and to explore the diagnostic and staging accuracy of computed tomography (CT) and magnetic resonance imaging (MRI) examinations.

**Methods:**

41 patients including 37 with UCS and 4 with CCS from July 2011 to September 2020 were enrolled in the study. Of the 37 UCS cases, 7 had CT images, 27 had MRI images, and 3 had both CT and MRI images. The Clinical data, CT or MRI imaging findings were analyzed. Diagnosis and staging accuracy of CT and MRI images were also analyzed.

**Results:**

Carcinosarcoma usually occurs in postmenopausal women (40/41), with the typical clinical symptom being vaginal bleeding (33/41). The CA125 degree was significantly different between the two invasion depth groups (*p* = 0.011). Most uterine carcinosarcomas showed unclear boundaries, uneven density, low or equal signal on T1WI, high or mixed signal on T2WI, uneven high signal on diffusion-weighted image (DWI), and mild enhancement. The diagnostic accuracies of CT and MRI for carcinosarcoma were 0% and 3.33%, respectively. The diagnostic accuracy for malignant tumors on CT and MRI was 50% and 83.33%, respectively.

**Conclusions:**

Carcinosarcoma lesions presented with huge mass filling in the cavity, and some presented with small polypoid lesions or endometrial thickening. Evaluation of lymph node metastasis is a significant challenge for imaging staging.

## Key points

- Some carcinosarcoma lesions presented with huge mass filling in the cavity.
- Some carcinosarcoma lesions presented with small polypoid lesions or endometrial thickening.
- Evaluation of lymph node metastasis is a significant challenge for imaging staging.

## Introduction

Carcinosarcoma of both the uterus and cervix, also known as malignant mixed Mullerian tumor, is a rare but highly malignant tumor. Among all uterine malignant tumors, the incidence of uterine carcinosarcoma (UCS) is less than 5% [1], but with a mortality rate of more than 15% [2], which is comparable to that of cervical carcinosarcoma (CCS) [3]. Preoperative diagnosis, differential diagnosis, and staging are of great significance for the possible operation or treatment management of these patients. Therefore, it is very helpful to analyze the imaging manifestations of carcinosarcoma.

Pathological biopsy takes a long time, and gets limited materials, which will cause bias to the result. Imaging examination is a good supplement. Ultrasonography is often used to detect lesions, while magnetic resonance imaging (MRI) and computerized tomography (CT) are commonly used for differentiation and stage assessment. Specifically, the first purpose of imaging examination is to find the lesions and predict the malignant degree and to recommend further examination or treatment. The second but important purpose is to show the location, the size, the blood supply and the invasion of surrounding tissues, which will help clinicians identify as surgical candidates and reduce operation time and surgical trauma. To date, the majority of these studies on UCS and CCS have been case reports. Few studies have addressed the diagnostic or staging accuracy of CT and MRI images. The purpose of our study was to analyze the clinical and imaging features of UCS and CCS, and to explore the diagnostic and staging accuracy of CT and MRI examinations.

## Materials and methods

The study was approved by the Institutional Review Board of our institution, and informed consent was waived.

### Patients

Patients with biopsy or pathologically confirmed UCS or CCS from July 2011 to September 2020 were identified from our pathology database and the Picture Archiving and Communication Systems. Patients who met the following criteria were excluded: (1) metastatic carcinosarcoma; (2) without enhanced CT or MRI images; (3) and history of anti-tumor therapy before imaging examination.

**Fig. 1:**
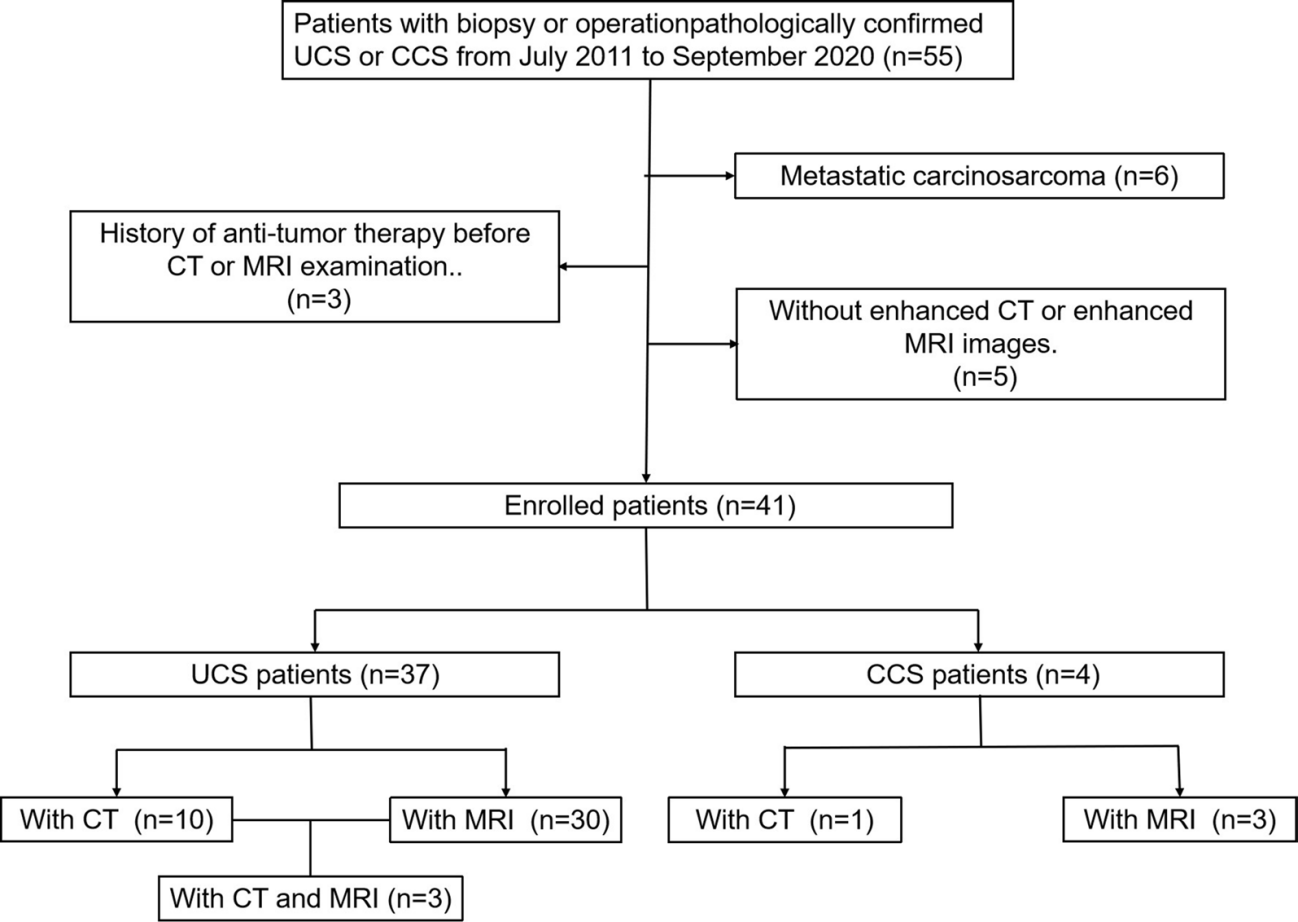
The flow chart of patient screening

Ultimately, 41 patients were enrolled, including 37 with UCS and 4 with CCS. Of the 37 UCS cases, 7 had CT images, 27 had MRI images, and 3 had both CT and MRI images. In four CCS cases, one had CT images, and three had MRI images. A flowchart is presented in Fig. 1.

### CT protocol

CT examinations were performed using a 64-row multi-detector CT scanner (Discovery CT750 HD CT Scanner, GE Healthcare, Waukesha, WI, USA). The parameters were as follows: tube voltage, 120 kVp; automatic tube current; rotation time, 0.5 s. A total of 70–100 mL of contrast medium (Ultravist 370, Bayer Schering Pharma, Berlin, Germany) was injected via a peripheral vein at a flow rate of 3.0–3.5 mL/s. Arterial phase CT images were obtained 10 s after attenuation of the abdominal aorta reached 150 Hounsfield units, while the venous phase images were obtained 30 s later. Coronal and sagittal images were reconstructed for each patient.

### MRI protocol

MRI images were acquired on a GE 3.0 T MRI scanner (Discovery MRI 750, GE Healthcare, Waukesha, Wisconsin, USA) with a torso phased array coil. The main MRI sequences were as follows: (1) axial T1-weighted imaging (T1WI): repetition time (TR), 600 ms; echo time (TE) 18 ms; slice thickness/section gap 4.0/1.0 mm; field-of-view (FOV) 280 × 280 mm; matrix 448 × 448. (2) Axial T2-weighted imaging (T2WI): TR 4000 ms; TE 85 ms; slice thickness/gap 4.0/1.0 mm; FOV 280 × 280 mm; matrix 384 × 384. (3) Sagittal T2WI: TR 4000 ms; TE 85 ms; slice thickness/gap 4.0/1.0 mm; FOV 280 × 280 mm; matrix 384 × 384. (4) Axial diffusion weighted image (DWI): *b* = 0, 800 s/mm^2^; TR 5400 ms; TE 85 ms; slice thickness/gap 4.0/1.0 mm; FOV 280 × 280 mm; matrix 192 × 192; number of excitation (NEX) 4. (5) Axial liver acquisition with volume acceleration (LAVA) dynamic contrast enhanced (DCE): TR 4.1 ms; TE 1.9 ms; slice thickness 3.0 mm; FOV 274 × 379 mm, matrix 288 × 208. The contrast medium (gadolinium-diethylenetriamine pentaacetic acid, Gd-DTPA; Magnevist, Bayer Schering Pharma, Berlin, Germany) with a dose of 0.2 mmol/kg was injected at a flow rate of 2.0 mL/s. This series included eight enhanced phases, with each phase lasting for 18 s. (6) Coronal delayed enhanced T1WI: TR 4.2 ms; TE 2.0 ms; slice thickness 3.0 mm; FOV 320 × 320 mm; matrix 320 × 320. Images were obtained 2 min after injection. (7) Sagittal delayed enhanced T1WI: TR 4.2 ms; TE 2.0 ms; slice thickness 3.0 mm; FOV 200 × 320 mm; matrix 260 × 320. Images were obtained 3 min after injection.

### Clinical analysis

Clinical data including age, sex, chief complaint, duration, childbearing history, and menopausal status were analyzed. The duration was divided into two groups according to median. Blood markers included alpha-fetoprotein (AFP), carcinoembryonic antigen (CEA), carbohydrate antigen 199 (CA199), carbohydrate antigen 153 (CA153), carbohydrate antigen 724, CA724, and carbohydrate antigen 125 (CA125). The stages were reviewed by an experienced expert, in accordance with International Federation of Gynecology and Obstetrics (FIGO) 2014 guidelines. The assessment was mainly based on the postoperative pathological results.

### Image analysis

Image findings included tumor margins, density/intensity, enhancement pattern and degree, invasion depth, adjacent tissue invasion, lymph node or peritoneal metastasis, imaging diagnosis and stage.

Enhancement pattern was classified as gradually, continuously and reduced at final, representing the enhancement degree in venous phase was higher, equal and lower than that in arterial phase. Enhancement degree in both CT and MRI images was classified as mild, moderate and marked, representing the enhancement degree of tumor was lower, equal and higher than that of muscles in venous phase. Tumor signal in T1WI and T2WI divided into low, equal, high and mixed signal, and mixed signal means that there were two or three signals. Imaging diagnosis were classified as carcinosarcoma, malignant tumor (carcinoma, sarcoma or other malignant tumors) and benign tumor (myoma or other benign tumors). UCS were staged according to FIGO 2014 staging guidelines for endometrial carcinoma. CCS were staged according to FIGO 2014 for carcinoma. Two radiologists diagnosed and staged the tumor, before and after being informed of the pathological results. Two radiologists (with 8 and 6 years of diagnosis experience in gynecology respectively) analyzed the CT and MRI findings independently. When there was a disagreement, the opinion of another senior radiologist (with 12 years of diagnosis experience in gynecology) will be the final standard.

Maximum diameter, maximum anteroposterior diameter (AP), and maximum endometrial thickness (ET) of the uterus or cervix were measured on sagittal images. Maximum and mean CT attenuations in the plain, arterial, and venous phases were also measured.

### Statistical analysis

Categorical variables were described as frequencies and percentages. Continuous variables were described as means and standard deviations or medians and quartiles. The Pearson *χ*^2^ test was used to evaluate the differences between the two groups. All statistical procedures were performed using the Statistical Package for the Social Sciences (SPSS 22.0, Chicago, IL, USA), and *p* < 0.05 was considered significant.

## Results

### Clinical characteristics

The preoperative clinical features of the 41 cases were reviewed (Table 1). The mean age of CCS patients was higher than that of UCS (*p* = 0.020). 29 (78.37%) patients with UCS and four (100%) patients with CCS suffered from vaginal bleeding. There was only one premenopausal woman with UCS. The symptom duration in 24 (64.86%) patients with UCS and 1 patient with CCS were less than 2 months. Thirteen (54%) patients with UCS presented with elevated CA125 levels. A total of 26 (79.27%) UCS patients and all CCS patients were at stage I. Bone metastasis occurred in one patient, lymph node metastasis occurred in 7 cases, and extensive abdominal cavity metastasis occurred in one patient. CA125 was significantly different between the two invasion depth groups (Table 2). An elevated CA125 indicates a deep invasion depth.

**Table 1:** Clinical features of UCS and CCS *n* (%)

Features	UCS (*n* = 37)	CCS (*n* = 4)	*p* value
Age, years	59.92 ± 7.40	69.25 ± 6.40	**0.020**
Childbearing history	3 (2, 3)	3.50 (2.25, 5.50)	0.190
*Chief complaint*			
Vaginal bleeding	29 (78.37)	4 (100)	0.569
Other	8 (21.62)	0	
*Duration*			
≤ 2 month	24 (64.86)	1 (15)	0.311
> 2 month	13 (35.14)	3 (75)	
*Postmenopausal*			
Yes	36 (97.29)	4 (100)	1.000
No	1 (2.7)	0	
*AFP*			
Elevated/normal	3/34	0/4	1.000
*CEA*			
Elevated/normal	0/37	0/4	–
*CA199*			
Elevated/normal	3/34	0/4	1.000
*CA125*			
Elevated/normal	13/24	0/4	0.385
*CA153*			
Elevated/normal	1/36	0/4	1.000
*CA724*			
Elevated/normal	1/36	0/4	1.000
*Invasion depth*			
≤ 1/2 myometrium	19 (51.35)	–	–
> 1/2 myometrium	18 (48.64)	–	
*Stage*			
IA/ IB	17/9	4	0.496
II	1	0	
IIIA/B/C1/C2	2/1/1/2	0	
IVB	4	0	

Bold value means *p* < 0.05

*UCS* uterine carcinosarcoma, *CCS* cervical carcinosarcoma, *AFP* alpha-fetoprotein, *CEA* carcinoembryonic antigen, *CA199* carbohydrate antigen 199, *CA125* carbohydrate antigen 125, *CA153* carbohydrate antigen 153, *CA724* carbohydrate antigen 724

**Table 2:** CA125 in different invasion depth group of UCS

	Invasion depth		*p* value
	≤ 1/2 myometrium	> 1/2 myometrium	
*CA125*			
Elevated	3	10	**0.011**
Normal	16	8	

Bold value means* p* < 0.05

*CA125* carbohydrate antigen 125

### CT and MRI features

Table 3 summarizes the CT characteristics of the 10 UCS cases. Most UCSs showed unclear boundaries, uneven density, and gradual or continuous mild enhancement. Arteries were visible in five cases, and capsule and pelvic effusion were invisible in most patients.

**Table 3:** CT characteristics of UCS *n* (%) (*n* = 10)

Characteristics	*N* (%)
*Margin*	
Clear	4 (40%)
Unclear	6 (60%)
*Density*	
Even	1 (10%)
Uneven	9 (90%)
*Enhancement degree*	
Mild	7 (70%)
Moderate	2 (20%)
Marked	1 (10%)
*Enhancement pattern*	
Gradually	5 (50%)
Continuously	5 (50%)
Reduced at final	0 (0)
*Capsule*	
Yes	2 (20%)
No	8 (80%)
*Blood supply arteries*	
Visible	5 (50%)
Invisible	5 (50%)
*Pelvic effusion*	
Visible	2 (20%)
Invisible	8 (80%)
Maximum diameter (mm)	73.77 ± 28.62
AP (mm)	68.67 ± 14.82
ET (mm)	43.64 ± 26.00
AP-ET	25.03 ± 16.94
ET/AP	0.61 ± 0.27
*CT attenuation of tissue components (Hu)*	
Max-plain	66.00 ± 13.99
Mean-plain	41.20 ± 5.75
Max-arterial	89.10 ± 17.41
Mean-arterial	66.00 ± 17.78
Max-venous	89.00 (77.75, 103.25)
Mean-venous	68.00 (53.75, 85.75)

*UCS* uterine carcinosarcoma, *AP* maximum anteroposterior diameter, *ET* maximum endometrial thickness, *Max-plain* maximum CT attenuation in plain phase, *Mean-plain* mean CT attenuation in plain, *Max-arterial* maximum CT attenuation in arterial phase, *Mean-arterial* mean CT attenuation in arterial phase, *Max-venous* maximum CT attenuation in venous phase, *Mean-venous* mean CT attenuation in venous phase

Table 4 summarizes the MRI characteristics of the 30 UCS cases. Similar to CT findings, most UCS showed unclear boundaries, low or equal signal on T1WI, high or mixed signal on T2WI, uneven high signal on DWI, and mild to moderate enhancement. Vascular washout effects were observed in eight cases.

**Table 4:** MRI characteristics of UCS *n* (%) (*n* = 30)

Characteristics	*N* (%)
*Margin*	
Clear	10 (33)
Unclear	20 (67)
*Capsule*	
Yes	4 (13)
No	26 (87)
*T1WI*	
Low signals	15 (50)
Equal signal	10 (33)
High signal	2 (7)
Mixed signal	3 (10)
*T2WI*	
Low signals	0
Equal signal	0
High signal	14 (47)
Mixed signal	16 (53)
*Vascular washout effect*	
Visible	8 (27)
Invisible	22 (74)
*Junctional zone*	
Uncontinuous	24 (80)
Continuous	6 (20)
*Pelvic effusion*	
Visible	5 (17)
Invisible	24 (80)
*Tumor hemorrhage*	
Visible	4 (13)
Invisible	26 (87)
*Homogeneous enhancement*	
Yes	3 (10)
No	27 (90)
*Enhancement pattern*	
Gradually	9 (30)
Continuously	14 (47)
Reduced at final	7 (23)
*Enhancement degree*	
Mild	13 (43)
Moderate	11 (37)
Marked	6 (20)
Maximum diameter (mm)	71.00 ± 29.54
AP (mm)	39.64 ± 18.67
ET (mm)	60.48 ± 16.60
AP-ET	20.83 ± 12.54
ET/AP	0.64 ± 0.21

*UCS* uterine carcinosarcoma, *AP* maximum anteroposterior diameter, *ET* maximum endometrial thickness

**Fig. 2:**
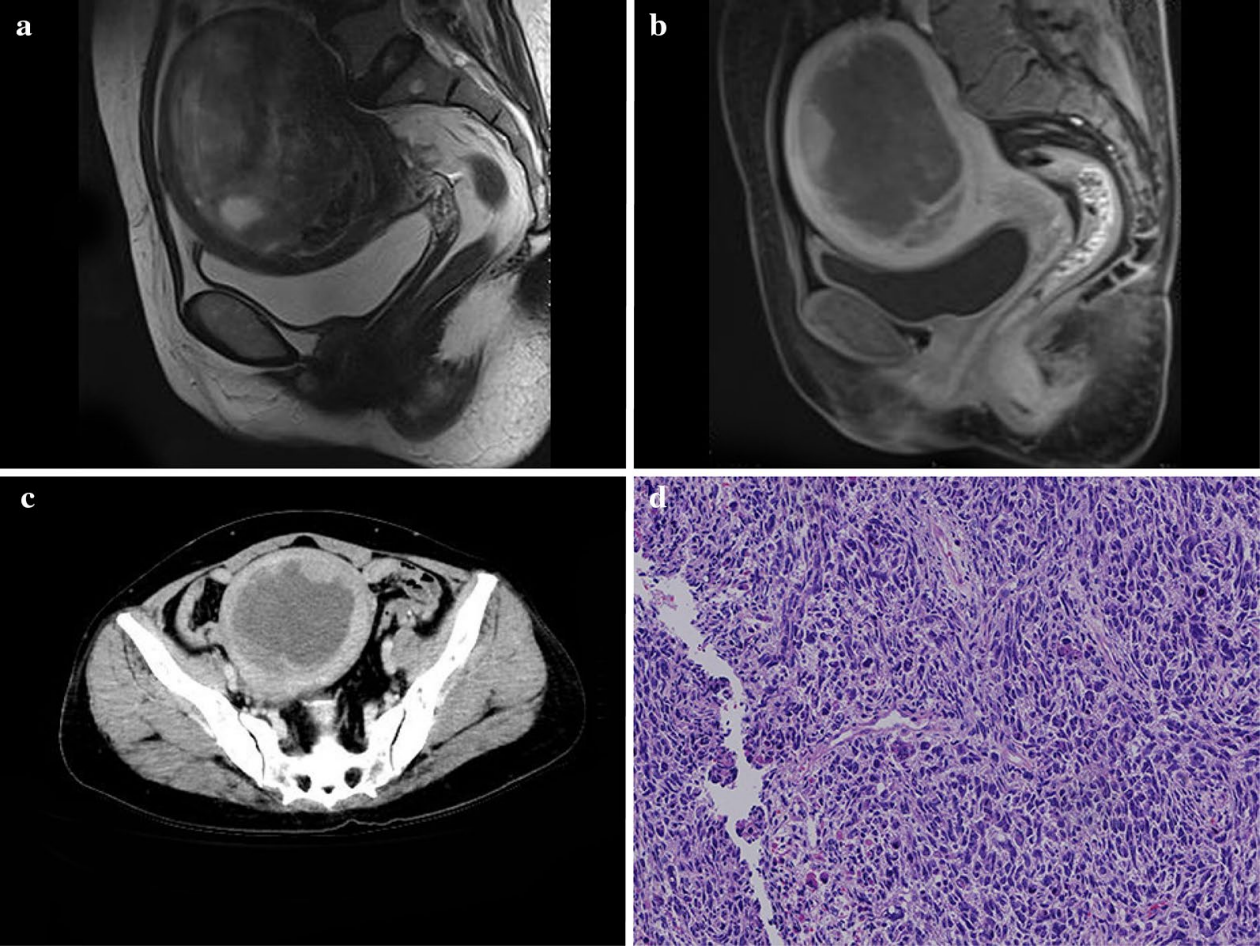
A 53-year-old woman with uterine carcinosarcoma (UCS). Sagittal T2WI magnetic resonance imaging (MRI) (**a**) showed a mixed signal tumor in the cavity complicated with intratumoral hemorrhage. Sagittal delayed enhanced T1WI (**b**) and axial computed tomography (CT) images in the venous phase (**c**) showed the marginal solid tissue component polyp-like enhancement with a central cystic component. The pathological image (**d**) showed uterine carcinosarcoma

The subjective overall manifestations of carcinosarcoma were summarized according to the CT and MRI findings. Most UCS lesions presented with huge mass filling in the cavity, with some presenting with small polypoid lesions or endometrial thickening. The masses showed three different density composition types: one type showed pure solid tissue components, one type showed large concentrated cystic components (Fig. 2), and the other type showed multiple small capsule components.

**Fig. 3:**
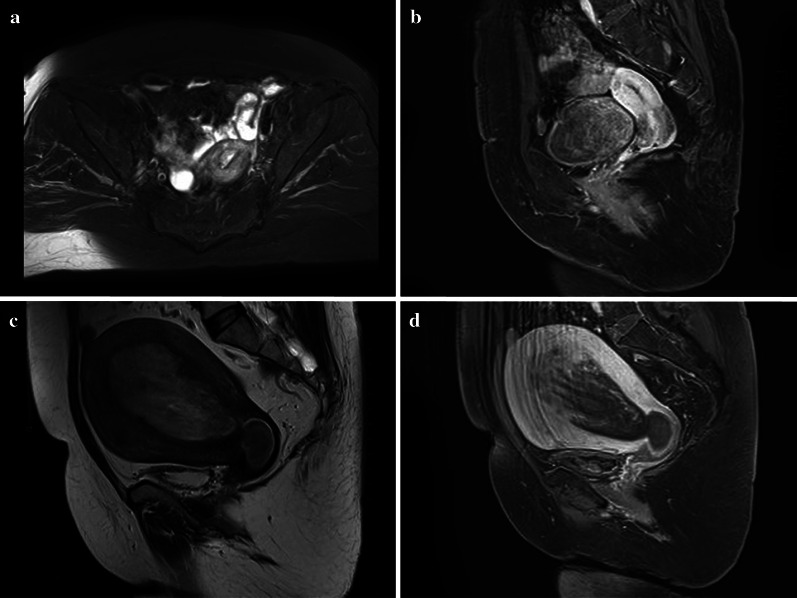
Images of uterine carcinosarcoma (UCS). Axial T2WI magnetic resonance imaging (MRI) (**a**) of a 63-year-old woman showed uterine fundus endometrial thickening with equal signal. Sagittal delayed enhanced T1WI (**b**) of the same patient showed a moderate enhancement. Sagittal T2WI MRI (**c**) of a 55-year-old woman showed a huge mixed signal tumor in the uterine and cervical cavity. Sagittal delayed enhanced T1WI (**d**) of the same patient showed a heterogeneous enhancement tumor

**Fig. 4:**
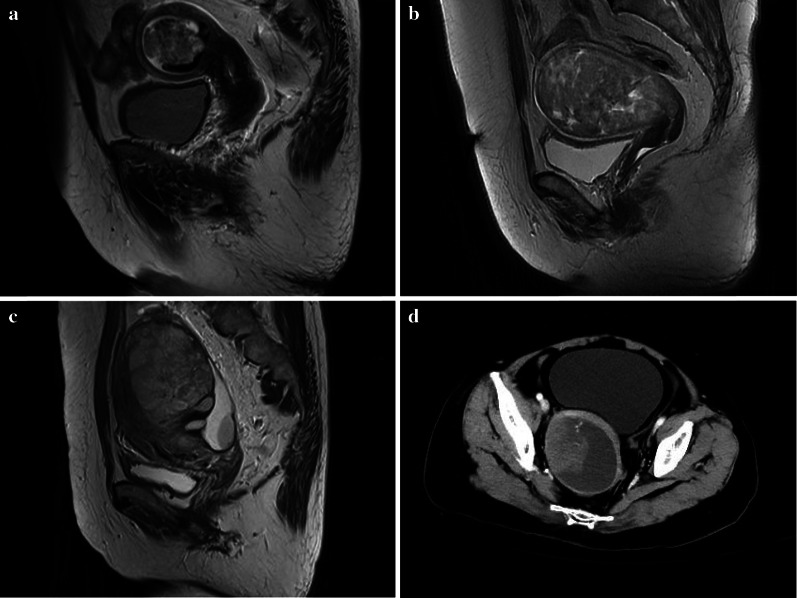
Images of uterine carcinosarcoma (UCS). Sagittal T2WI magnetic resonance imaging (MRI) of a 64-year-old woman (**a**) showed a mixed signal tumor in the cavity with multiple small round or irregular cystic areas in the internal tumor. Sagittal T2WI MRI of a 66-year-old woman (**b**) showed a mixed signal tumor in the cavity with many gap-like cystic areas. Sagittal T2WI MRI of a 49-year-old woman (**c**) showed a high signal tumor in the cavity with many oval cystic areas. Axial computed tomography (CT) images in the venous phase (**d**) of a 66-year-old woman showed a tumor mainly composed of cystic components. The tissue components showed nipple-like enhancement with a marginal cystic component

The pure solid tissue components showed heterogeneous density/intensity, gradual and mild enhancement, and persistent moderate enhancement at the same time. The large concentrated cystic components may be unobvious on plain CT scan but obvious after enhancement. This was because the cystic components were composed of complex mucus components. The gelatinous liquid in the pathology also confirmed this finding. The marginal solid tissue showed a polyp-like enhancement with a central concentrated cystic component, and the central solid tissue showed cord-like or nipple-like enhancement with a marginal concentrated cystic component. Multiple small capsule components showed multiple small round or irregular cystic areas in the internal tumor with unclear boundaries to solid tissue or many gap-like cystic areas (Figs. 3, 4). The solid tissue components of the last two types showed a gradual mild enhancement.

### The diagnostic and staging accuracy of CT and MRI

The diagnostic accuracy for carcinosarcoma on CT and MRI were 0% and 3.33%, respectively; while for malignant tumors on CT and MRI, it was 50% and 83.33%, respectively.

The staging accuracies of CT and MRI were 60% and 70%, respectively (Table 4). Three cases were overestimated in CT images, two of which occurred in lymph node metastasis and one case in invasion depth. In one case, lymph node metastasis was underestimated on CT images. Five cases were overestimated in MRI images, two of which occurred in lymph node metastasis, two cases in interstitial infiltration, and one case in adnexal invasion. Four cases had underestimated staging in MRI images: two occurred in lymph node metastasis, one in interstitial infiltration, and one in invasion depth (Table 5).

**Table 5:** Diagnosis and staging accuracy of CT and MRI images

	CT	MRI
*Diagnosis*		
Carcinosarcoma	0 (0.00%)	1 (3.33%)
Malignant tumor	5 (50.00%)	27 (83.33%)
Benign tumor	5 (50.00%)	2 (13.33%)
*Staging*		
Accuracy	6 (60.00%)	21 (70.00%)
Overestimated	3 (30.00%)	5 (16.67%)
Underestimated	1 (10.00%)	4 (13.33%)

### Image features of CCS

**Fig. 5:**
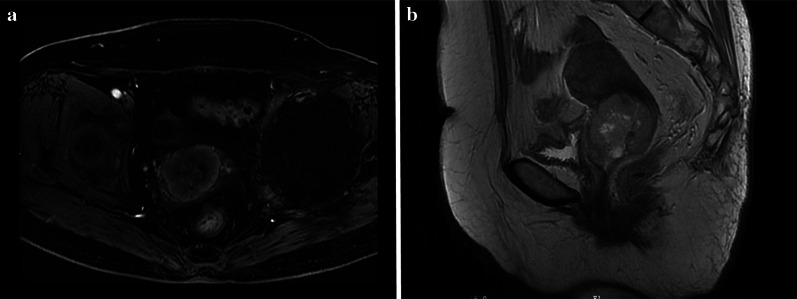
A 70-year-old woman with cervical carcinosarcoma (CCS). Sagittal T2WI magnetic resonance imaging (MRI) (**a**) showed a mixed signal tumor in the cavity. Axial delayed enhanced T1WI (**b**) showed a heterogeneous mild enhancement tumor

One patient with CCS had a CT image, and three patients had MRI images. One patient showed cervical endometrial thickening with mild enhancement. Three cases showed a cervical mass with cystic and solid tissue components with heterogeneous enhancement. Tumors presented mixed or low signal on T1WI and mixed or high signal on T2WI (Fig. 5). All CCSs were present at stage I.

## Discussion

This retrospective study included 41 patients with UCS or CCS who underwent CT or MRI examinations. We analyzed the clinical and imaging features, compared CA125 degrees in two invasion depth groups, and explored the diagnostic and staging accuracy of CT and MRI examinations. Most importantly, we summarized the case of carcinosarcoma. The summary and classification of the subjective overall manifestations will improve the sensitivity and accuracy of diagnosis, filling the gaps in the literature.

Carcinosarcoma usually occurs in postmenopausal women; the incidence of carcinosarcoma in premenopausal women is exceedingly rare. A 25 years old woman with CCS has been reported [4–6]. The typical clinical symptoms are vaginal bleeding; other symptoms include vaginal discharge, abdominal mass, abdominal pain, or no obvious symptoms. Unlike ovarian carcinosarcoma [5], the majority of UCS and CCS are at an early stage without peritoneal metastasis. However, even though carcinosarcoma is in stage I, the 5-year survival rate is still less than 50% [6]. It was previously reported that invasion depth and clinical stage were independent risk factors for prognosis [7]. The elevated CA125 level indicates a deeper invasion depth with poor prognosis in the present study, which is consistent with the findings of a previous study [8, 9].

In this study, 53% of carcinosarcomas presented with mixed signals, which is consistent with the complicated histopathological components. Carcinosarcoma has both cancer and sarcoma components, and sometimes even various multiple sarcomas. Necrosis also contributes to mixed signals [10]. A carcinosarcoma with a necrotic region if > 10% may have a poor prognosis [1]. Tumors typically present low or equal signals on T1WI and high or mixed signals on T2WI; high signal areas on T1WI may represent hemorrhage [10, 11]. A tumor mainly characterized by a low signal on T2WI is considered a malignant tumor, except for carcinosarcoma [12]. Mild and moderate enhancement is another important differential point to distinguish carcinosarcoma from other malignant tumors [10, 11].

The damaged intimal line, damaged junction zone and the obvious high signal on DWI suggests malignant tumors and benign tumors such as endometrial polyps or uterine leiomyoma will not be considered [13]. Malignant tumors will also be considered with elevated CA125. The signifcant point of distinguish carcinosarcoma and carcinoma are the inhomogeneity on T2WI. As summarized in our results, the carcinosarcoma mass showed three different density composition types, which is consistent with the complicated histopathological components [14]. The presence of high signal areas on T1WI is a rare but highly specific feature of carcinosarcoma. The carcinosarcoma often shows progressive or persistent mild or moderate enhancement, while the carcinoma often shows mild enhancement in the early stage and decrease in the late stage [10] (Table 6).

**Table 6:** Differential diagnostics of carcinosarcoma

Characteristics	Benign tumors	Carcinoma	Carcinosarcom
Damaged intimal line	−	+	+
Damaged junctional zone	−	+/−	+/−
CA125	−	+/−	+/−
The presence of high signal areas on T1WI	−	−	+/−
Inhomogeneity T2WI	−	+	+ +
Diffusion limited DWI	−	+	+ +
Enhancement pattern	Most gradually/continuously	Most reduced at final	Most gradually/continuously
Enhancement degree	Most mild	Most mild	Most mild/moderate

MRI has a higher soft tissue resolution than CT and can clearly show the boundaries of the endometrium, myometrium, and interstitium. CT has a limited value in the diagnosis of carcinosarcoma at an early stage. Both CT and MRI can show invasion of adjacent organs or peritoneal metastasis when the tumor is at an advanced stage, while MRI is considered the first-line modality after lesions are detected.

Although carcinosarcoma contains both epithelial and mesenchymal components, the cancerous component is the driving force for tumor deterioration. The risk factors and clinical behavior of carcinosarcoma are similar to those of endometrial carcinoma [15]. According to the International Federation of Gynecology and Obstetrics, carcinosarcoma is a special variant of endometrial carcinoma or cervical carcinoma [16]. Therefore, we staged UCS and CCS according to FIGO 2014 for carcinoma. Stages were mistakenly estimated in 13 cases using 40% CT images and 30% MRI images. It has been noticed that seven of them occurred in lymph node metastasis. It is difficult to judge lymph node metastasis based on the size, shape, or density of both CT and MRI.

This study has several limitations. First, this is a study of a small series, and most patients either underwent CT or MRI, but not both. Although we can't compare the performance of the two examinations, we can still summary of image features and get the trend that MRI is superior to CT in the accuracy of diagnosis, but not in staging. Small sample size may also cause deviation in the comparison of clinical characteristics data. A larger sample of data is needed to confirm these findings. Second, a small number of patients were diagnosed by biopsy pathological examination, and the stages of some metastatic patients were evaluated by CT and MRI images, which may have led to some bias.

## Conclusions

Carcinosarcoma lesions presented with huge mass filling in the cavity, with some presenting with small polypoid lesions or endometrial thickening. The mass showed three different density composition types. Most uterine carcinosarcomas showed unclear boundaries, uneven density, low or equal signal on T1WI, high or mixed signal on T2WI, uneven high signal on DWI, and mild enhancement. When these manifestations are present, the diagnosis of carcinosarcoma should be considered. Evaluation of lymph node metastasis is a significant challenge for staging.

## Data Availability

Please contact author for data requests.

## Abbreviations

AFP: Alpha-fetoprotein
CA125: Carbohydrate antigen 125
CA153: Carbohydrate antigen 153
CA199: Carbohydrate antigen 199
CA724: Carbohydrate antigen 724
CCS: Cervical carcinosarcoma
CEA: Carcinoembryonic antigen
CT: Computed tomography
DCE: Dynamic contrast enhanced
DWI: Diffusion-weighted image
FIGO: International Federation of Gynecology and Obstetrics
FOV: Field-of-view
LAVA: Liver acquisition with volume acceleration
MRI: Magnetic resonance imaging
NEX: Number of excitations
T1WI: T1-weighted imaging
T2WI: T2-weighted imaging
TE: Echo time
TR: Repetition time
UCS: Uterine carcinosarcoma
